# Notch Signaling in Neuroendocrine Tumors

**DOI:** 10.3389/fonc.2016.00094

**Published:** 2016-04-14

**Authors:** Judy S. Crabtree, Ciera S. Singleton, Lucio Miele

**Affiliations:** ^1^Department of Genetics, Louisiana State University Health Sciences Center, New Orleans, LA, USA; ^2^Stanley S. Scott Cancer Center, Louisiana State University Health Sciences Center, New Orleans, LA, USA

**Keywords:** Notch, neuroendocrine tumors, carcinoid, pNET, small-cell lung cancer, SCLC

## Abstract

Carcinoids and neuroendocrine tumors (NETs) are a heterogeneous group of tumors that arise from the neuroendocrine cells of the GI tract, endocrine pancreas, and the respiratory system. NETs remain significantly understudied with respect to molecular mechanisms of pathogenesis, particularly the role of cell fate signaling systems such as Notch. The abundance of literature on the Notch pathway is a testament to its complexity in different cellular environments. Notch receptors can function as oncogenes in some contexts and tumor suppressors in others. The genetic heterogeneity of NETs suggests that to fully understand the roles and the potential therapeutic implications of Notch signaling in NETs, a comprehensive analysis of Notch expression patterns and potential roles across all NET subtypes is required.

## Introduction

Notch has been studied for many years in the context of cancer, and over the years, the signaling pathways involved have become clearer. However, as these pathways are elucidated, the complexity of Notch signaling is revealed as well. It is now known that in addition to canonical Notch signaling where the activated Notch receptors can play tumor suppressive roles in some cancer types and oncogenic roles in others, non-canonical signaling is also active in some cell types and impacts signaling through phosphatidylinositol 3′ kinase (PI3K)/Akt, mTOR, NF-kB, and β-catenin (1–6). In neuroendocrine tumors (NETs), these same signaling pathways, as well as hairy enhancer of split 1 (Hes-1)/achaete–scute complex-like 1 (ASCL-1), have been shown to impact tumorigenesis *via* Notch signaling (7–14). Translationally, many of these pathways have modulatory or inhibitory drugs in development that may be applied to the treatment of NETs, but the role of Notch signaling in this diverse set of tumors must be more clearly defined. Here, we outline current knowledge of the Notch canonical and non-canonical signaling in NETs as well as highlight understudied areas.

## Canonical Notch Signaling

**Figure 1:**
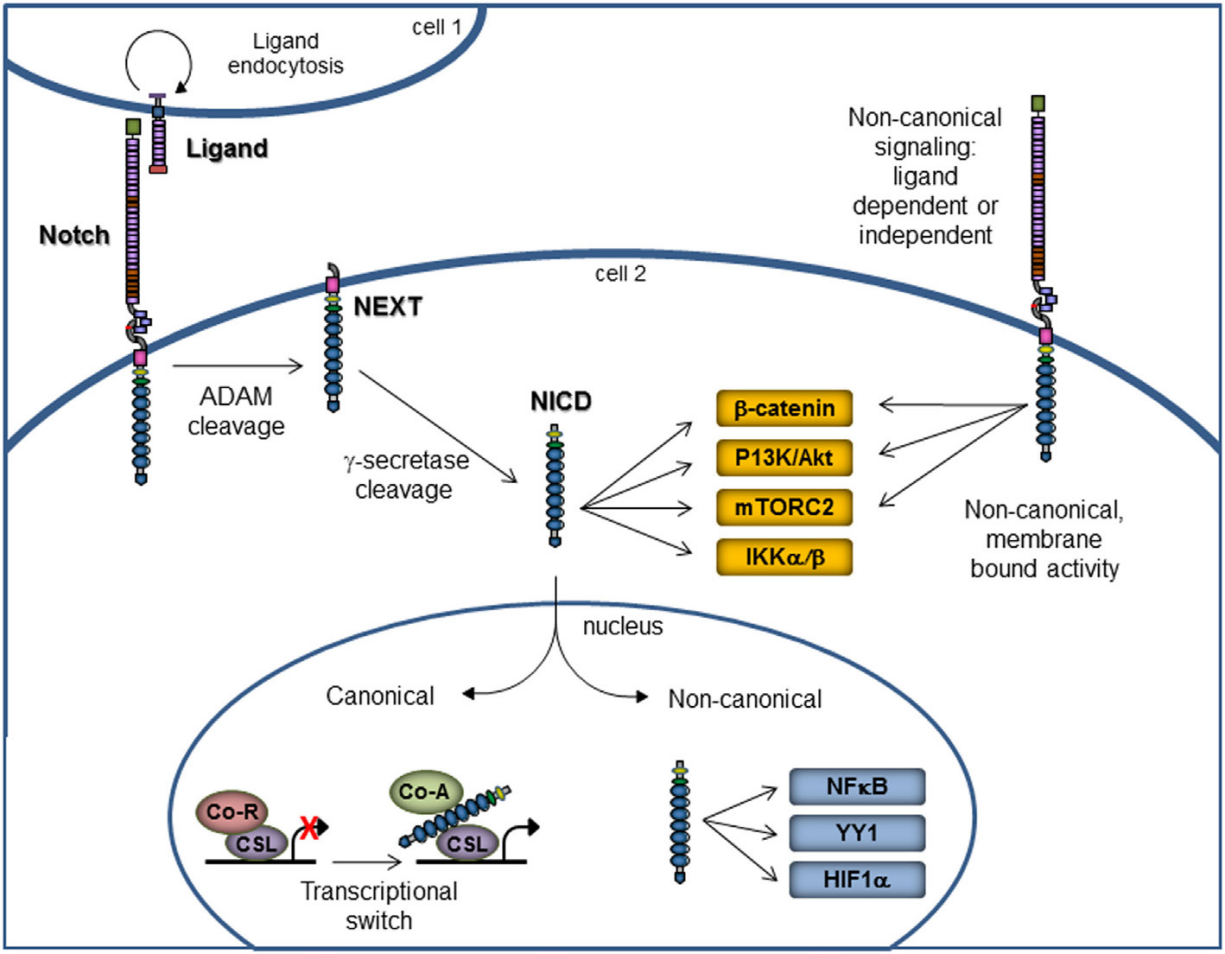
**Notch canonical and non-canonical signaling**. Notch signaling *via* the canonical pathway is on the left portion of the figure. Membrane-bound Notch receptor is activated by binding with ligand on a neighboring cell, which results in cleavage by ADAM metalloproteases, followed by cleavage with γ-secretase. These cleavage events release the NICD, which then enters the nucleus to affect the gene transcription. The non-canonical signaling pathway is on the right portion of the figure and illustrates that non-canonical signaling may occur either in the presence or absence of ligand. Further, the signaling may occur *via* the membrane-bound, uncleaved Notch receptor or *via* the NICD. Non-canonical Notch signaling is independent of CSL and allows for interaction with PI3K/AKT/mTORC2, Wnt/β-catenin, IKKα/β, NFκB, YY1, and HIF1α pathways at the cytoplasmic and/or nuclear level. Abbreviations: ADAM, a disintegrin and metalloprotease; NEXT, Notch extracellular truncation; NICD, Notch intracellular domain; Co-R, corepressor; Co-A, coactivator.

The Notch signaling pathway has long been recognized as a central player in cellular processes, such as proliferation, stem cell maintenance, and differentiation during both embryonic and adult development. Notch signaling is evolutionarily conserved across species and relies on the presence of the Notch receptor binding *in trans* to ligand present on a neighboring cell. In canonical signaling, ligand binding promotes the intracellular cleavage of the receptor by metalloproteases to release the active form of Notch, the Notch intracellular domain (NICD), which translocates into the nucleus and binds to transcription factor *C*BF-1/*S*uppressor of Hairless/*L*AG-1 (CSL), also known as RBP-Jκ, to activate expression of Notch-responsive genes (Figure 1) (15–18).

**Figure 2:**
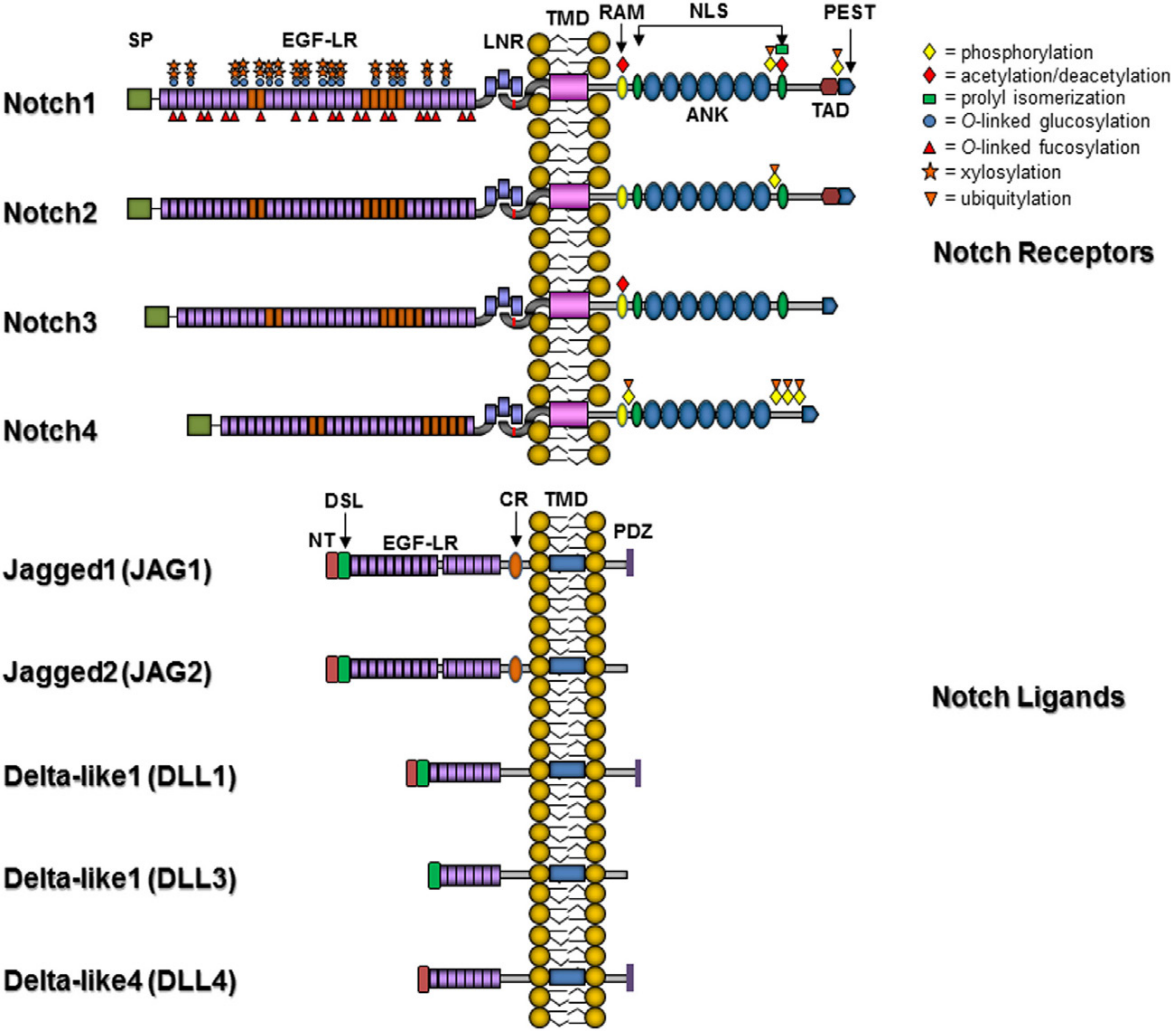
**Notch receptors and ligands**. Abbreviations: SP, signal peptide; EGF-LR, epidermal growth factor-like repeats; LNR, Lin-Notch repeat; TMD, transmembrane domain; RAM, RBP-Jκ association module 23; NLS, nuclear localization signal; ANK, ankyrin/CDC10 repeat; TAD, transactivation domain; PEST, proline/glutamic acid/serine/threonine-rich motif; PDZ, PSD-95/Dlg/ZO-1 domain; CR, cysteine-rich domain; DSL, Delta/Serrate/Lag2 domain; NT, N-terminal domain. Posttranslational modifications are indicated by symbols: yellow diamonds, phosphorylation; red diamonds, acetylation/deacetylation sites; green square, prolyl isomerization site; blue circle, O-linked glucosylation; red triangle, O-linked fucosylation; orange star, xylosylation; and inverted orange triangle, ubiquitylation.

The number of Notch receptor genes varies by species, with *Drosophila* containing one Notch receptor, *Caenorhabditis elegans* having two redundant receptors, and mammals containing four Notch receptors, Notch1–4. The Notch receptors contain an extracellular domain that includes multiple epidermal growth factor (EGF)-like repeats that are essential for ligand binding and vary in length across the four mammalian receptors. The intracellular portion of Notch is critical for transmission of cellular signals and contains an RBP-Jκ association module (RAM) domain, a nuclear localization signal (NLS), a seven ankyrin repeat (ANK) domain, and a transactivation domain that contains conserved proline/glutamic acid/serine/threonine-rich (PEST) motifs (Figure 2). The ligands for Notch receptors are varied and have been extensively reviewed in Ref. (17). In mammals, Notch ligands include Delta-like 1 (DLL1) and Delta-like 4 (DLL4), homologous to *Drosophila* Delta, along with Jagged 1 (JAG1) and Jagged 2 (JAG2), homologous to *Drosophila* Serrate. Delta-like 3 (DLL3) may be an inhibitory ligand that sequesters Notch receptors in the cytoplasm (19). These ligands are responsible for the majority of known canonical Notch signaling effects and like Notch have multiple EGF-like repeats in their extracellular domains. These type 1 transmembrane proteins all contain an N-terminal sequence that along with the DSL (Delta/Serrate/Lag2) motif and the first two EGF-like repeats are required for ligand-receptor binding. In contrast to the DLL ligands, the Jagged ligands have almost twice the number of EGF repeats and also contain an additional cysteine-rich region. The intracellular portion of all Notch ligands lacks major homology with the exception that some, but not all, ligands contain multiple lysine residues and a C-terminal PDZ (PSD-95/Dlg/ZO-1) domain. Stimulation of the Notch signaling pathway ultimately results in the transcriptional activation of a discrete set of genes by the formation of a Notch transcriptional complex at the promoters of target genes or within enhancer or superenhancer regions (20). This complex includes the NICD, which translocates into the nucleus and displaces a corepressor complex to bind to CSL, first through interaction with the RAM domain followed by the ANK domain. This binding mediates a transcriptional switch to activate transcription from promoters containing CSL binding sites (GTGGGAA) and is dependent on the formation of a ternary complex, including one of mastermind-like 1–3 (MAML1–3) coactivators (21), and ski-interacting protein (SKIP) (22–24). This tertiary complex in turn recruits additional coactivator proteins such as the histone acetyltransferases CREB-binding protein (CBP)/p300 (25) or p300/CBP-associated factor (PCAF)/GCN5 (26). In the absence of NICD, CSL actively represses transcription from Notch target genes, and a large number of repressor complex components have been identified, including histone deacetylases (27), silencing mediator for retinoid and thyroid hormone receptor/nuclear receptor corepressor (SMRT/NCoR) (28), SMRT/HDAC-1-associated repressor protein/Msx2-interacting nuclear target (SHARP/MINT)/SPEN (29, 30), CSL-interacting corepressor (CIR) (31), hairy and enhancer of split 1 (Hes1) (32), hairy-related transcription factor 1 (HRT1) (32), c-Jun N-terminal kinase (JNK)-interacting protein-1 (JIP1) (33), and lysine-specific demethylase 5A/retinoblastoma-binding protein 2 (KDM5A/RBP2) (34), among others.

Endogenous NICD is relatively short lived. It is transiently present at Notch target promoters in response to ligand and degrades rapidly in part due to phosphorylation of the C-terminal PEST domain by cyclin C/cyclin-dependent kinase (CDK) 8 (35). Phosphorylation of the PEST domain is followed by ubiquitinylation and subsequent proteasome degradation (36, 37). Furthermore, phosphorylation near the ankyrin repeats of NICD has also been reported to negatively regulate Notch transcriptional activation by interfering with the formation of the Notch activation complex (38). On the contrary, phosphorylation of NICD by glycogen synthase kinase 3β (GSK3β) occurs in a region C-terminal to the ANK repeats and increases the stability of NICD (39). Fringe enzymes (*N*-acetylglucosaminyltransferases) influence binding affinity between Notch receptors and specific EGF-like repeats by glycosylation (40–42). Glycosylation by fringe results in elongation of O-linked fucose residues on particular EGF-like repeats in Notch and prevents Notch activation by Jagged ligands, but not by Delta-like ligands (43, 44). Another form of O-linked carbohydrate modification on Notch receptors is *O*-glucose, which is attached to serine residues by the *O*-glucosyltransferase Rumi/POGLUT1 (45). O-glycosylated Notch EGF-like repeats can be further modified by the addition of xylose by glucoside xylosyltransferase (GXYLT)1 and GXYLT2 and xyloside xylosyltransferase, resulting in negative regulation of Notch signaling (46–48). Acetylation by CBP/p300 and PCAF/GCN5 enhances NICD stability (49, 50), as does prolyl isomerization by Pin1 (51), whereas deacetylation of NICD and histones by Sirtuin 1 (SIRT1) inhibits Notch signaling (52, 53). These posttranslational modifications may also be Notch paralog specific. For example, the Notch4 intracellular domain (Notch4-ICD) is the only Notch paralog with sites for AKT phosphorylation. Notch4-ICD that is phosphorylated by AKT then binds to 14-3-3ζ and accumulates in the cytoplasm, thereby blocking Notch4 gene regulation (54).

## Non-Canonical Notch Signaling

Notch signaling can also occur in a non-canonical fashion that is independent of CSL and can be ligand dependent or independent (Figure 1) (1, 55). While canonical Notch signaling is well studied and crucial in various cellular processes as mentioned above, the known mechanisms of non-canonical signaling are limited but known to occur in cancer and some immune system cells (1). Non-canonical Notch pathways present an interesting new avenue of study and may reveal new targets for therapeutic intervention in the translational setting.

Notch signaling that occurs in the absence of CSL acts upon various cellular pathways that are involved in cancer and immune system responses. One such pathway is the Wnt/β-catenin pathway. The Wnt/β-catenin pathway is a conserved pathway that regulates cell pluripotency and cell fate decisions in development and postnatal life. Aberrant functions or mutations in β-catenin have been associated with a number of cancers and other human diseases. Non-canonical Notch signaling converges upon the Wnt/β-catenin signaling pathway and results in an ­antagonistic interaction between Notch signaling and Wnt/β-catenin (6, 55, 56). This interaction disrupts the regulation of developmental and disease processes in a context (tissue)-dependent manner (55) and leads to negative regulation of Wnt signaling by altering the level of active β-catenin activity (3). High levels of membrane-bound Notch are associated with lower levels of active β-catenin, suggesting an inverse relationship between the two as well as a potential negative regulation of β-catenin by Notch (55). One example of this crosstalk is the loss of Notch1 in the epidermis of mice, which results in activated Wnt/β-catenin signaling and the formation of hyperplasia and cancer – both of which can be reversed by the introduction of exogenous NICD (57). In cervical cancer cells, Notch1 activates NF-kB *via* IKKα, which migrates to the nucleus in a Notch-dependent fashion (58); similarly, nuclear IKKα mediates the effect of Notch1 in ER^+^ breast cancer cells, whereby Notch1 activates ERα-dependent transcription in an IKKα-dependent fashion in the absence of estrogen (59).

In the immune system, non-canonical Notch signaling is involved in the activation and proliferation of CD4^+^ T cells as well as in the tumor-promoting effects of interleukin-6 (IL-6) (1, 60). These events rely on NF-κB and demonstrate crosstalk with other cellular pathways in the absence of canonical Notch signaling. Studies by the Osborne lab have established that even in the absence of CSL, activation and proliferation of CD4^+^ T cells does occur and requires Notch1 playing a major role in the signature CBM complex (CARMA1, MALT1, and BCL10) (61) by which T cells become activated through NF-κB (62). IL-6 has also been demonstrated as a Notch target gene in breast cancer cells, with the Notch-dependent activation of IL-6 reliant on IKKα/β function, but not on the canonical NF-κB signaling cascade (2). Furthermore, the Notch1 intracellular domain was shown to activate a non-canonical signaling cascade *via* mTORC2 and Akt as a means of transmitting extracellular nutrient sensing cues to promote cell survival (4, 63).

## Neuroendocrine Tumors – Enteropancreatic

Carcinoids and NETs are a heterogeneous group of tumors that arise from the neuroendocrine cells of the GI tract, endocrine pancreas, and the lung (addressed separately below). Enteropancreatic NETs are slow growing tumors that can be associated with symptoms caused by peptide hormone release (“functional” NETs) and pose a significant threat due to high metastatic potential. The annual incidence of enteropancreatic NETs ranges from 2 to 5 per 100,000 patients in the United States and recent analyses suggest that this incidence will rise in the coming years (56, 64–66). The median overall survival (OS) for metastatic pancreatic and small bowel NETs is 24 and 56 months, respectively (65). Typically, enteropancreatic NETs are classified based on observable factors, such as anatomical site, histology, grade, level of differentiation, and hormone secretion, but due to the heterogeneous nature of the disease, this classification has led to confusion in both research and clinical settings. It is now recognized that NETs must be subdivided into pancreatic and non-pancreatic subgroups to reduce heterogeneity in clinical trials and patient care management. For a recent review on clinical management of NET patients, see Ref. (67). Progression-free survival (PFS), instead of OS, has become a frequently used endpoint in clinical trial design, and the degree of tumor differentiation has been noted as a key indicator of outcome. Differentiated tumors have a much better prognosis than poorly differentiated tumors, which can have a 5-year survival of less than 4% (66).

The only curative enteropancreatic NET treatment is surgery, and this is only effective if the tumors are removed prior to metastasis. Somatostatin analogs (SSAs), VEGF pathway inhibitors, mTOR inhibitors, and peptide receptor radionuclide therapy (PRRT) are currently in clinical practice and/or clinical trials, and have demonstrated moderate success. SSAs, such as octreotide, lanreotide, and pasireotide, have been used to control symptoms as a result of hormone hypersecretion (carcinoid syndrome) in these patients. More recently, SSAs have been noted to have antiproliferative effects on well or moderately differentiated NETs (68, 69), as reported for metastatic midgut NETs in the PROMID trial (70), and in pancreatic, midgut, or hindgut NETs in the CLARINET trial (71). Radiolabeled SSAs are also used in PRRT for a localized anticancer therapy in patients with inoperable or highly metastatic NETs. The first prospective, randomized trial, the NETTER-1 trial, is underway to compare radiolabeled [^177^Lu-DOTA^0^, Tyr^3^]octreotate with the standard of care high-dose octreotide LAR in patients with inoperable, somatostatin receptor-positive metastatic midgut NETs (NCT01578239) with the primary endpoint of PFS. mTOR inhibitors, specifically everolimus or RAD001, showed efficacy the RADIANT-3 trial in patients with advanced pancreatic NETs when compared to placebo. Median PFS was 11 months compared to 4.6 months with placebo (72). The results of the RADIANT-3 trial led to FDA approval for everolimus for the treatment of advanced pancreatic NETs in 2011. Finally, the oral tyrosine kinase inhibitor sunitinib was studied in a prospective trial in patients with advanced, well-differentiated pancreatic NETs. PFS was 11.6 months in the sunitinib group compared to 5.5 months in the placebo arm (73). As with the RADIANT-3 trial, the increase in PFS resulted in FDA approval of sunitinib for advanced, well-differentiated pancreatic NETs. For a comprehensive review of all carcinoid and NET clinical trials, see Ref. (74). Given the heterogeneity of NETs, a better understanding of drug function, mechanism, and optimal patient group selection will guide future therapeutic strategies and clinical trials.

The genetics of NETs may also play a role in treatment ­development and selection. Genetic syndromes account for 15–20% of NETs, including multiple endocrine neoplasia type 1 and type 2 (MEN1 and MEN2), von Hippel–Lindau syndrome (VHL), neurofibromatosis type 1 (NF1), and tuberous sclerosis complex (TSC), but the remaining 80–85% of NET are considered sporadic. In an attempt to understand driving genetic mutations that result in pancreatic NETs, Jiao et al. (75) performed exome sequencing of 10 pancreatic NETs to identify mutated genes. This resulted in the identification of somatic mutations in a number of cancer genes, including MEN1, DAXX, ATRX, a number of genes involved in the mTOR pathway, and to a lesser extent TP53. A subsequent study by Banck et al. of 48 well-differentiated, small intestinal NETs (carcinoids) were analyzed by whole exome sequencing, and somatic mutations were identified in many cancer-associated genes, including FGFR2, MEN1, HOOK3, EZH2, MLF1, CARD11, VHL, NONO, SMAD 1, FANCD2, and BRAF (76). Analysis of 55 well-differentiated small intestinal NETs in a separate study identified 1230 genes with somatic variants (77), only 21 of which were in common with the Banck study. Further, upon comparison with the Jiao et al. study (75), only 17 genes with somatic mutations were in common with pancreatic NETs (77). These studies highlight the heterogeneity of NET tumors and reinforce that this group of tumors needs to be carefully studied, subgrouped, and analyzed to account for heterogeneity in terms of site of origin, level of differentiation, and underlying driver mutations. Interestingly enough and despite the somewhat disparate results, all of these studies have highlighted the putative role of chromatin remodeling, perhaps in concert with Notch signaling, in the etiology of enteropancreatic NETs.

## Neuroendocrine Tumors – Pulmonary

Pulmonary NETs comprise a separate, diverse set of NETs that are classically described as falling on a continuum from well-differentiated typical carcinoid (TC), to less differentiated atypical carcinoid (AC), to highly malignant and poorly differentiated small-cell lung carcinoma (SCLC), and large cell neuroendocrine carcinoma (LCNECs) (78). The distinction between these different tumor types is based on the WHO clinicopathological criteria of the mitotic index (number of mitoses per 2 mm^2^, usually equal to 10 high power fields). The mitotic index of TC is <2, AC is 2–10, whereas SCLC and LCNECs have mitotic indices >10 (78). Immunohistochemistry using neuroendocrine markers, such as synaptophysin, chromogranin A, and neural cell adhesion molecule (NCAM), are used to confirm neuroendocrine origin and define SCLC from non-SCLC. Although present at a much lower incidence than other pulmonary NETs, mixed pulmonary NETs can also form as a heterogeneous, combination of tumors consisting of mixtures of SCLC and LCNEC, or SCLC and non-SCLC with neuroendocrine differentiation (78).

The incidence of pulmonary NETs is low, roughly 1.57/100,000 individuals. TCs comprise 1–2%, and ACs make up only 0.1–0.2% of pulmonary tumors, whereas SCLC and LCNET make up 20 and 1.6–3%, respectively. The OS is 92–100% for TCs and 61–88% for ACs, whereas the higher grade SCLC and LCNET have a much poorer prognosis with OS as low as 5% (79). Treatment options for pulmonary NETs are limited. The only curative therapeutic option for TC and AC is surgery. These tumors do not respond well to chemotherapy and exhibit a response rate as low as 22% (80). SCLC and LCNEC are rarely treated with surgery because patients often present initially with advanced stage disease. The first-line treatment is chemotherapy (typically etoposide combined with carboplatin), with initial response rates as high as 90%, but the majority of tumors recur and are resistant to further treatment (80). Studies in SCLC have evaluated the mTORC1 inhibitor everolimus in combination with standard of care chemotherapies cisplatin and etoposide, but dose-limiting toxicities and modest clinical efficacy suggest that this therapeutic combination is unlikely to be pursued (81). SSAs have been studied in a small number of clinical trials on pulmonary NETs, and the efficacy of these drugs on TC and AC is still under debate. The RADIANT-2 trial included enteropancreatic NETs as well as pulmonary TC and ACs treated with placebo plus octreotide LAR or everolimus plus octreotide LAR. Subgroup analyses from this study found a median PFS of 5.6 months for the few TC and AC patients who received only the octreotide LAR (82). A further trial is now open in Europe, called the LUNA trial which is a prospective, randomized, open-label, and three-arm design to study advanced lung (TC and AC) and thymic NET response to pasireotide LAR, everolimus, or both in combination (NCT01563354). In a phase II study in patients with relapsed or refractory SCLC, treatment with sunitinib was poorly tolerated and resulted in minimal gain in PFS (83). In addition to mTOR inhibitors, tyrosine kinase inhibitors, such as imatinib, have been studied in pulmonary NETs with disappointing results (84).

As with enteropancreatic NETs, the genetics of pulmonary NETs have also been explored in recent years. Genome-wide studies have been performed (85–88) to identify copy number alterations, point mutations, and changes in the transcriptome of SCLC. These studies identified copy number changes in the Myc family of oncogenes as well as potential driver mutations in genes such as TP53, RB1, CREBBP, EP300, MLL, and the SOX family. A separate study conducted whole-genome sequencing of 110 SCLC and identified biallelic inactivation of TP53, RB1, CREBBP, EP300, TP73, and RBL1/2, as well as inactivating mutations in Notch family genes in 25% of cases (88, 89). Exome sequencing of pancreatic and lung NET cell lines was reported earlier this year by Boora et al. (90). This study demonstrated a similar spectrum of mutant genes as those found in primary tumors, including TP53, RB1, EP300, and Notch, but also TSC2, GNAS, KDR, STK11, and APC. Interestingly enough, this analysis of lung and pancreatic NET cell lines did not identify DAXX, ATRX, or MEN1, and the authors suggested that the genetic signatures of these cell lines were not consistent with primary tumors and data from these cell lines should be interpreted with caution (90).

## Notch Signaling and NETs

The NICDs of all Notch proteins are potentially oncogenic, and deregulated Notch signaling has been shown in many solid tumors, including breast (18, 91, 92), cervical (93), endometrial (94), esophageal (95), gastric carcinoma (96), glioma (97), head and neck (98), hepatocellular (99), lung (100), medulloblastoma (101), melanoma (102), mesothelioma (7), ovarian (103), pancreatic (104), prostate (105), renal (106), and rhabdomyosarcoma (107). Additionally, Notch signaling is deregulated in hematological malignancies as well, including T-cell acute lymphoblastic leukemia (T-ALL) (108, 109), Hodgkin lymphomas (110), some acute myeloid leukemias (111), B-cell chronic lymphoid leukemia (112), and multiple myeloma (113). These observations suggest that dysregulated Notch signaling prevents differentiation and leads to malignancies in some of these cancers, while in others, the oncogenic role of Notch is likely due to inhibition of apoptosis.

The abundance of literature on the Notch signaling pathway is a testament to the complexity of this process in different cellular environments. This is especially true with a heterogeneous tumor group such as NETs. NETs remain significantly understudied with respect to molecular mechanisms of pathogenesis, and particularly Notch signaling. Mechanistically, Notch may contribute to carcinogenesis by inhibiting differentiation, promoting cellular proliferation, and/or inhibiting apoptosis, yet few studies have examined these endpoints in NETs. The relatively few studies published to date have focused primarily on the expression and function of Notch1. Contrary to the many tissue types discussed above, these studies suggest a tumor suppressive function for Notch1 in neuroendocrine lineage cells. This is consistent with role of Notch in *Drosophila* neurogenesis, where it prevents neuroectodermal cell differentiation toward the neuronal lineage. In *Drosophila* embryos, loss of Notch results in a “neurogenic” phenotype, where differentiation toward the neuronal lineage is uncontrolled (114, 115).

It is plausible that loss of Notch1 signaling would allow NET cells to acquire or maintain a partially differentiated neuroendocrine phenotype while retaining the ability to proliferate. For example, recent studies (11, 12, 116–119) report that Notch1 signaling is minimal or absent in pulmonary TC and AC and gut carcinoids. Yet these same cancers express high levels of human achaete–scute homolog 1 (hASH1), a basic helix-loop-helix transcription factor regulated by Notch signaling. Shida et al. propose that the aberrant expression of hASH1 may reflect the decreased differentiation and maturation of gastrointestinal NETs, suggesting that since hASH1 is not degraded temporospatially as it should be by Notch1-activated Hes1 and Hes5, the cells are arrested at an early stage of differentiation (119).

Studies in BON1 cells transiently overexpressing Notch1 NICD resulted in growth suppression, dose-dependent increases in Hes1, and a decrease in NET markers, confirming the tumor-suppressor function of Notch1 signaling in pancreatic NETs. In contrast, immunohistochemistry for Notch1, Hes1, Hey1, pIGF1R, and FGF2 antibodies on a tissue microarray of 120 well-differentiated NETs arising from the pancreas (*n* = 74), ilium (*n* = 31), and rectum (*n* = 15) demonstrated elevated Notch1 expression in 100% rectal, 34% of pancreatic, and 0% of ileal NETs, and Hes1 expression in 64% of rectal, 10% of pancreatic, and 0% of ileal NETs (120), exhibiting significant variability in Notch1 signaling across different tissue types. Furthermore, in the lung, Notch signaling can either promote or inhibit lung cancer, depending on tumor types. For example, Notch1 ­activation is thought to promote the growth of NSCLC but inhibit that of SCLC (121, 122). Studies on the expression of other Notch receptors and ligands in NETs are few. Notch3 is known to play a tumor suppressive role in medullary thyroid carcinoma (123), but the role of Notch signaling in other NETs is understudied. Notch3 expression is decreased in SCLC compared to non-tumor lung tissue by immunohistochemistry, suggesting that Notch3 is involved in tumor suppression in SCLC (124). This may be the result of deregulated Notch functions in cell fate decisions that determine differentiation toward the epithelial Clara, ciliated, and pulmonary neuroendocrine cell lineages (125). In mouse models with allelic series deletion of Notch1–3, all three Notch receptors are required in an additive manner to regulate the abundance of neuroendocrine cells, whereas only contribution from Notch2 is required for Clara/ciliated cell development in the lung (126). There is limited information on Notch4 or the ligands involved in canonical Notch signaling in NETs. A comprehensive analysis of Notch expression patterns across all NET subtypes is required to fully understand the variability and redundant functions of Notch receptors and ligands.

Additional complexities arise in the form of transcriptional coactivators and corepressors that bind to NICD to regulate gene expression, as a growing body of evidence suggests that Notch behaves as an oncogene or a tumor suppressor depending on cellular context. For example, Notch1 is an oncogene in most systems, but in skin (127), some squamous epithelia (128), vasculature (129, 130), and potentially NETs, it behaves as a tumor suppressor (15). It is well established that the NICD binds to CSL, MAML, SKIP, and p300 to activate transcription of Notch-responsive genes *via* canonical Notch signaling. Similarly, the presence of varied corepressors in the absence of NICD also regulates transcription in specific ways that may be underappreciated. SMRT (28), SIRT (53), and histone lysine demethylase (LSD1) (131), among others [reviewed in Ref. (132)] have all been identified as corepressors of Notch/CSL signaling. Notch activator and repressor complexes have also been implicated in the epigenetic regulation of Notch signaling. Many Notch coactivators and corepressors are histone acetyltransferases, histone demethylases, histone methyltransferases, etc. and as a part of a complex with CSL, actively remodel the chromatin at Notch-responsive target genes, providing an additional layer of reversible regulation (34). Chromatin sites accessible to Notch NICDs are also influenced by other transcriptional regulators that can act as cofactors or inhibitors (133–135). A recent report by Liefke et al. (34) demonstrates that the histone demethylase KDM5A/RBP2 is a key component of the CSL repressor complex.

An additional layer of complexity is produced by paralog-specific effects. While in theory, all Notch receptors signal through CSL, they are not completely redundant, and there are instances in which their functions are not only independent but opposite. In NSCLC, Notch1 and Notch2 have opposite effects on Akt (136). Notch2 has been described as a tumor suppressor in breast cancer cell lines (137), while Notch1, 3, and 4 are uniformly oncogenic in the breast. The mechanism of these paralog-specific effects is unclear. They may involve “private” non-canonical signals, such as the inhibitory role of Notch4 on SMAD (138) or the stimulatory role of Notch1 on NF-κB (139). The oncogenic activity of Notch4 in the mouse mammary gland does not require CSL and is therefore completely or at least partially non-canonical (140). Alternatively, paralog-specific effects may be explained by quantitative differences in signal intensity. For instance, constitutively activating mutations in Notch1 and Notch2 are equally oncogenic in a subset of triple negative breast cancer (TNBC) (141), despite the fact that Notch2 has been described as a tumor suppressor in TNBC cell lines (137). In other words, variable signal intensity (the number of NICD molecules available as a result of overproduction or reduced degradation) may dictate different phenotypic consequences. This may be achieved perhaps by selective activation of chromatin sites with different affinity for Notch NICDs, similar to the well-known dose-dependent effects of p53, or by a combination of canonical and non-canonical effects that depend on NICD abundance. The role of paralog-specific effects has not been well characterized in NETs and is an area in need of further study.

Targeted therapies to modulate the Notch signaling pathway have been under development for several years, including neutralizing antibodies, decoy ligands, blocking peptides, natural compounds, and γ-secretase inhibitors [reviewed in Ref. (18)]. The Notch 2/3 neutralizing antibody tarextumab inhibits tumor growth in mice not only in a variety of epithelial tumors but also in SCLC xenograft tumors (142). This suggests that either Notch2 or Notch3 inhibition can have therapeutic activity in SCLC cells or that non-cell autonomous effects on tumor stroma mediated by Notch2/3 inhibition are responsible for this effect. An interesting way of exploiting decreased Notch signaling therapeutically consists of targeting Notch ligands that are frequently overexpressed even in tumors with low canonical Notch signaling. An especially effective strategy for NETs was pioneered in SCLC, which frequently expresses high levels of DLL3. DLL3 can function as a Notch inhibitor, by retaining Notch receptors into the cytoplasm or by *cis*-inhibition. A DLL3 mAb conjugated with a toxic chemotherapeutic agent was highly effective in preclinical models of SCLC. However, the naked mAb had no therapeutic activity, suggesting that DLL3 inhibition alone is not a viable therapeutic strategy in SCLC (14).

## Conclusion

The role of Notch signaling in NETs remains incompletely understood, but the careful and systematic study of Notch signaling in these tumors may reveal unique therapeutic possibilities by leveraging drugs in development or approved for other indications. Paralog-specific effects may be prominent in these tumors, and there is some preliminary evidence that Notch1 and perhaps Notch3 act as a tumor suppressors in some of NETs but not in others. Further, expression data suggest that there may be significant heterogeneity among NETs in terms of expression of Notch receptors and target genes. The roles of Notch2, Notch4, and Notch ligands, if any, are understudied and remain unclear. It is known that DLL3 is expressed in some SCLC and is a useful targeting antigen for therapeutic immunotoxins, but the role of other ligands is unknown. The significant genetic heterogeneity of NETs suggests that individual molecular subtypes may have to be studied separately to dissect the roles of Notch signaling components and their potential therapeutic implications.

## Author Contributions

JC wrote the manuscript and generated the figures. CS wrote portions of the manuscript. LM wrote portions of the manuscript and edited the final version and figures.

## Conflict of Interest Statement

The authors declare that the research was conducted in the absence of any commercial or financial relationships that could be construed as a potential conflict of interest.
